# Differences in insectivore bird diets in coffee agroecosystems driven by obligate or generalist guild, shade management, season, and year

**DOI:** 10.7717/peerj.12296

**Published:** 2021-10-27

**Authors:** Julie A. Jedlicka, Stacy M. Philpott, Martha L. Baena, Peter Bichier, Thomas V. Dietsch, Laney H. Nute, Suzanne M. Langridge, Ivette Perfecto, Russell Greenberg

**Affiliations:** 1 Department of Biology, Missouri Western State University, Saint Joseph, Missouri, USA; 2 Environmental Studies Department, University of California, Santa Cruz, Santa Cruz, California, United States; 3 Instituto de Investigaciones Biológicas, Universidad Veracruzana, Xalapa, Veracruz, Mexico; 4 Migratory Bird Program, U.S. Fish and Wildlife Services, Carlsbad, California, USA; 5 Paulson Ecology of Place Initiative, Wellesley College, Wellesley, Massachusetts, United States; 6 School for Environment and Sustainability, University of Michigan-Ann Arbor, Ann Arbor, Michigan, United States; 7 Smithsonian Migratory Bird Center, Smithsonian Conservation Biology Institute, National Zoological Park, Washington, District of Columbia, USA

**Keywords:** Avian diet, Interspecific competition, Natural history, Neotropical migrant, Niche partitioning, Omnivore, Polyculture, Resident, Obligate insectivore, Generalist insectivore

## Abstract

Neotropical shade-grown coffee systems are renowned for their potential to conserve avian biodiversity. Yet, little is known about food resources consumed by insectivorous birds in these systems, the extent of resource competition between resident and migratory birds, or how management of shade trees might influence diet selection. We identified arthropods in stomach contents from obligate and generalist insectivorous birds captured in mist-nets at five coffee farms in Chiapas, Mexico between 2001–2003. Overall stomach contents from 938 individuals revealed dietary differences resulting from changes in seasons, years, and foraging guilds. Of four species sampled across all management systems, Yellow-green Vireo (*Vireo flavoviridis*) prey differed depending on coffee shade management, consuming more ants in shaded monoculture than polyculture systems. Diets of obligate and generalist resident insectivores were 72% dissimilar with obligate insectivores consuming more Coleoptera and Araneae, and generalist insectivores consuming more Formicidae and other Hymenoptera. This suggests that obligate insectivores target more specialized prey whereas generalist insectivores rely on less favorable, chemically-defended prey found in clumped distributions. Our dataset provides important natural history data for many Nearctic-Neotropical migrants such as Tennessee Warbler (*Leiothlypis peregrina; N = 163*), Nashville Warbler (*Leiothlypis ruficapilla; N = 69*), and Swainson’s Thrush (*Catharus ustulatus; N = 68*) and tropical residents including Red-legged Honeycreepers (*Cyanerpes cyaneus; N = 70*) and Rufous-capped Warblers (*Basileuterus rufifrons; N = 56*). With declining arthropod populations worldwide, understanding the ecological interactions between obligate and generalist avian insectivores gives researchers the tools to evaluate community stability and inform conservation efforts.

## Introduction

Coffee farms cover large areas of mid-elevation landscapes in much of the northern Latin American tropics and, in many areas where deforestation has been rampant, coffee agroecosystems provide one of the few habitats with trees (*e.g.*, Rice & Ward, 1996). Traditional shade coffee farms with high vegetation complexity, abundant epiphytes, and an emergent tree layer support an abundance of woody plants (Philpott et al., 2008a; Tadesse, Zavaleta & Shennan, 2014), insects (Klein et al., 2002; Philpott et al., 2008a; Jha & Vandermeer, 2010), birds (Philpott et al., 2008a; Arendt et al., 2020), bats (Williams-Guillén & Perfecto, 2010), and other mammals (Gallina, Mandujano & Gonzalez-Romero, 1996; Caudill, DeClerck & Husband, 2015). Shade coffee systems were recognized in the late 1990s as important habitat for Neotropical migratory bird species (Greenberg, Bichier & Sterling, 1997; Greenberg et al., 1997, 2000). However, bird species richness declines with increasing intensification of coffee management (Moguel & Toledo, 1999; Philpott et al., 2008a) that can result in reduced tree richness and density, lower canopy height and/or lack of emergent trees, fewer epiphytes, absence of fruit, nectar, and arthropod resources, and changes in the density of understory plants (Greenberg et al., 1997; Cruz-Angon & Greenberg, 2005; Philpott et al., 2008a; Bakermans et al., 2012; Smith et al., 2018). Consequently, coffee farm management can be defined along a continuum, with higher levels of associated biological diversity in shaded farms with high levels of planned biodiversity.

Bird species foraging in coffee habitats are mostly obligate or generalist insectivores, the latter consuming fruit along with insects and sometimes referred to as omnivores. The seasonal influx of Neotropical migrants and to a lesser extent Austral migrants may result in resource competition for arthropods with resident bird species (Poulin & Lefebvre, 1996). Changes in bird abundance, richness, and composition are important from a conservation standpoint, but also because birds can provide ecosystem services in agroforestry systems. In particular, insectivorous birds can provide pest control in coffee agroecosystems (Greenberg et al., 2000; Van Bael et al., 2008; Johnson et al., 2009; Philpott et al., 2009; Milligan et al., 2016). Birds may limit populations of economically important coffee insect pests such as coffee berry borers (*Hypothenemus hampei*; Kellermann et al., 2008; Karp et al., 2013; Martínez-Salinas et al., 2016) and coffee leaf miners (*Leucoptera coffella*; Borkhataria, Collazo & Groom, 2006). If the bird foraging frequency and maneuvers differ depending on shade tree identity (Bakermans et al., 2012), shade tree composition (Dietsch, Perfecto & Greenberg, 2007), foraging strata (Wunderle & Latta, 1998; Jedlicka et al., 2006; Dietsch, Perfecto & Greenberg, 2007), or the bird species present on a farm (*e.g.*, Wunderle & Latta, 1998, Newell et al., 2014), this may alter what birds are consuming, and subsequently the relative impact of birds on arthropods in the coffee and canopy vegetative layers.

Birds use various food resources and different foraging strata in coffee farms, but limited information is available concerning how bird foraging behavior and diets might shift with changes in management of coffee farms. In shade coffee farms in the Dominican Republic, Wunderle & Latta (1998) examined the foraging behavior of 19 species of birds and found that all species used the canopy layer, with eight species foraging exclusively in the canopy and only one species foraging primarily in the coffee layer. Both invertebrates and nectar were important food resources in the canopy, whereas invertebrates were the main food item in the coffee layer. Jedlicka et al. (2006) found that migrants were more likely to forage in the canopy where arthropods were more abundant than the coffee understory, potentially causing the resident birds to forage more in the coffee layer during the dry season. Bakermans et al. (2012) found that more birds classified as upper canopy foragers were found at shade coffee farms with taller trees and more understory vegetation. Newell et al. (2014) examined the foraging behavior and tree species selection of three species of canopy foliage-gleaning migrants and found that both foraging height and foraging maneuvers differed among species, but all three species foraged in *Inga* spp. trees more than expected based on relative abundance. These studies contribute to our knowledge of how birds use foraging strata and substrates in shade coffee farms, but not how differences in shade coffee management might influence foraging behavior and diets across the bird community. Dietsch, Perfecto & Greenberg (2007) compared bird foraging behavior and diets in two coffee farms in Mexico across two seasons and noted that foraging maneuvers used by birds varied with tree species, management type, and season. Across both seasons and management types, >60% of foraging observations were for arthropods, with relatively greater use of fruit in the wet season and nectar in the dry season. Although this study documented that birds took lepidopteran prey more frequently during the wet than dry season, no other specifics about the prey items were documented. Thus, although we outline what is known about how birds forage in the canopy and coffee layer of shade coffee farms, and some information about how this behavior differs with coffee management, we know less about how bird diets differ across seasons and with differences in how coffee farms are managed.

Although bird communities change with coffee management intensification (Wunderle & Latta, 1998; Perfecto et al., 2004; Johnson, Kellermann & Stercho, 2010), the specific details of bird diets, such as which arthropod prey insectivorous birds are consuming in different coffee habitats or in different seasons, and how changes in foraging behavior may influence diets are not well understood. Moreover, recent research suggests that divergent migrant foraging behavior still results in broad overlap in dietary components among wood warblers (Kent & Sherry, 2020) so studies documenting dietary items are necessary to understand food web dynamics. Understanding avian diets is critical for informing variables chosen in ecological modelling of foraging (Railsback & Johnson, 2011) and evaluating conservation potential of working landscapes (Sánchez-Clavijo, Bayly & Quintana-Ascencio, 2020). Some investigators have examined bird diets indirectly using exclosure studies to examine how excluding birds from coffee plants or shade trees influences arthropod populations (Greenberg et al., 2000; Philpott et al., 2004; Borkhataria, Collazo & Groom, 2006; Karp et al., 2013). Others have observed bird foraging to infer diet items (Wunderle & Latta, 1998), and still others have used gut content analysis to examine bird diets (Sherry et al., 2016; Kent & Sherry, 2020). More recently, researchers have used metabarcoding techniques (termed molecular scatology) to identify specific pest species consumed by birds foraging in coffee and other agricultural systems (Karp et al., 2013; Crisol-Martínez et al., 2016; Jedlicka, Vo & Almeida, 2017). However, despite the development of molecular scatology techniques, there are benefits to more traditional dietary approaches, especially in quantifying dietary contents, which is often not possible with use of molecular techniques that focus on the presence or absence of particular diet items.

To advance our understanding of how coffee management and season influence bird diets, we examined the diets of obligate and generalist species of insectivorous birds in coffee agroecosystems in Chiapas, Mexico. Our specific objectives were to determine (1) the arthropod prey in the diets of obligate and generalist insectivorous birds, (2) how diets might change from year to year, with season, or with the changing community dynamics between resident and migratory bird species, and (3) how diets might differ depending on how coffee agroecosystems are managed. We examined differences and overlap of arthropod dietary contents among bird species during the dry and wet seasons and over multiple years. Interspecific interactions can change from year to year, so multi-year studies are critical for understanding the shifting relationships among bird species.

## Materials & methods

### Study sites

Our study area encompassed ~5,000 ha that was originally tropical lower montane and premontane moist and subtropical lower montane and montane wet forest. The area was developed for coffee production in the early 1900s, and the landscape is now ~90% coffee agroecosystems and ~10% small (<2 ha) forest fragments (Philpott et al., 2008b). The topography of the region is rugged, steep, and mountainous. All farms are located between 950–1,150 m asl and receive ~4,200–5,000 mm of rain annually. The region is strongly seasonal, with a wet season that typically lasts from May to October and a dry season from November to April.

We worked at five sites in three coffee farms (300-ha) in the Soconusco region of southwest Chiapas, Mexico, including *Finca* Belén, *Finca* Irlanda, and *Finca* Hamburgo. The five sites represented a gradient of coffee management intensity and differed in the structure of canopy vegetation and coffee plants (Mas & Dietsch, 2003; Philpott, Perfecto & Vandermeer, 2006). Based on a common classification scheme (Moguel & Toledo, 1999), TP stands for traditional polycultures, CP are commercial polycultures, and SM is a shaded monoculture dominated by *Inga* spp. The five sites were: (1) Belén Rustic (TP1; 15°, 15′N, 92°, 22′W), (2) Belén Production (CP1; 15°, 15′N, 92°, 23′W), (3) Irlanda Restoration (TP2; 15°, 10′N, 92°, 20′W), (4) Irlanda Production (CP2; 15°, 11′N, 92°, 20′W), and (5) Hamburgo (SM; 15°, 10′N, 92°, 19′W). TP1 is an area where the natural canopy vegetation has largely been maintained, and understory shrubs were replaced with coffee plants. TP2 is an area of active forest restoration where some native and some productive trees, including *Eriobotria japonica* (loquot), *Manguifera indica* (mango), *Citrus sinensis* (orange), *Persea americana* (avocado), and *Terminalia amazonia* (a timber species) have been planted with the intent of restoring the area to forest. All other sites included varying densities and diversities of shade trees, but the most common canopy trees were *Inga* spp., *Alchornea latifolia*, *Trema micrantha*, and *Conostegia xalapensis*. Vegetation data collected in 1998 at all study sites (Mas & Dietsch, 2003) and at all sites except TP2 (Philpott, Perfecto & Vandermeer, 2006) from 2000 to 2002 revealed differences between sites in canopy cover, tree species richness, vegetation structural depth and complexity, coffee bush density and height, and overall management intensity, with a general trend of increasing coffee management intensification such that TP1 < TP2 < CP2 < CP1 < SM (Table S1). All sites were certified organic with the exception of SM.

We collected data during three wet seasons and three dry seasons from 2001 to 2003. In our study sites, the typical dry season period falls between early November and late April while the typical wet season falls between early May and late October. Dry season data (when Neotropical-Nearctic migratory and resident birds were present) were collected from 11 to 25 January 2001, 29 November to 13 December 2002, and 12 to 24 March 2003. Wet season data (when Austral migratory and resident birds were present) were collected from 5 to 28 June 2001, 23 to 28 July 2002, and 19 to 28 July 2003. Although the dates differ by year, none of the sample dates fell exactly at the start or end of the typical dry or wet season for our sites.

### Bird diets

We captured birds in mist-nets placed in each study site. Mist-nets (four to six 12-m, 30-mm mesh nets) were placed in the same location on consecutive days and moved after 2–5 d if capture activity dropped noticeably. Nets were placed at ground level between rows of coffee. All mist-netting was conducted between 08:30 and 12:00; on a few overcast days, mist-nets remained open until as late as 15:00. Research methods were approved by the University of Michigan’s Animal Care and Use Program Permit Number 7499 and Mexico’s Secretaría de Medio Ambiente y Recursos Naturales Permit Number 10092.

We processed all arthropod-consuming birds captured that could handle the emetic used to obtain diet samples (exceptions included small to medium-sized hummingbirds, other than Violet Sabrewings (*Campylopterus hemileucurus*), and raptors). However, if too many birds were captured to process them in a timely manner, we generally prioritized the most frequently captured species (Table 1) to ensure sufficient sample sizes.

**Table 1:** Bird species sampled including family, common name, scientific name (Pyle & DeSante 2018), number of independent samples, migratory status, and foraging strata and guild following Greenberg et al. (1997), unless otherwise noted in the source.

Family	Common name	Scientific name	Sample number	Strata*	Guild**	Migratory status^^^	Production^^^^	Source^#^
Trochilidae	Violet Sabrewing	*Campylopterus hemileucurus*	10	U	N/I	R	TP CP SM	DE
Furnariidae	Olivaceous Woodcreeper	*Sittasomus griseicapillus*	11	T	I	R	TP	
Tyrannidae	Greenish Elaenia	*Myiopagis viridicata*	7	C	F/I	R	TP CP	D
	Ochre-bellied Flycatcher	*Mionectes oleagineus*	23	U	F/I	R	TP CP	
	Yellow-olive Flatbill	*Tolmomyias sulphurescens*	20	C	F/I	R	TP CP	
	Tropical Pewee	*Contopus cinereus*	4	C	I	R	CP	
	Least Flycatcher	*Empidonax minimus*	12	S	I	M	TP CP	
	Dusky-capped Flycatcher	*Myiarchus tuberculifer*	4	C	F/I	R	TP CP	
Vireonidae	Blue-headed Vireo	*Vireo solitarius*	4	C	F/I	M	TP CP	
	Warbling Vireo	*Vireo gilvus*	9	C	I	M	TP CP	
	Yellow-green Vireo	*Vireo flavoviridis*	64	C	F/I	AM	TP CP SM	B
	Lesser Greenlet	*Pachysylvia decurtata*	7	C	I	R	TP CP	D
Troglodytidae	Spot-breasted Wren	*Pheugopedius maculipectus*	18	S	I	R	TP CP SM	
	Banded Wren	*Thryophilus pleurostictus*	6	S	I	R	CP	D
	Rufous-and-white Wren	*Thryophilus rufalbus*	24	S	I	R	TP CP	D
	Cabanis’s Wren	*Cantorchilus modestus*	25	S	I	R	TP CP SM	
	House Wren	*Troglodytes aedon*	39	S	I	R	TP CP SM	
	White-breasted Wood Wren	*Henicorhina leucosticta*	7	U	I	R	TP	
Turdidae	Orange-billed Nightingale-Thrush	*Catharus aurantiirostris*	18	U	F/I	R	TP CP	
	Swainson’s Thrush	*Catharus ustulatus*	68	U	F/I	M	TP CP	P
	Clay-colored Thrush	*Turdus grayi*	12	U	F/I	R	TP CP	P
Passerellidae	Prevost’s Ground Sparrow	*Melozone biarcuata*	13	S	F/I	R	CP SM	
Parulidae	Ovenbird	*Seiurus aurocapilla*	16	U	I	M	TP CP	
	Worm-eating Warbler	*Helmitheros vermivorum*	10	U	I	M	TP CP	
	Black-and-white Warbler	*Mniotilta varia*	13	T	I	M	TP CP	
	Tennessee Warbler	*Leiothlypis peregrina*	163	C	F/I	M	TP CP	
	Nashville Warbler	*Leiothlypis ruficapilla*	69	C	F/I	M	TP CP	
	Magnolia Warbler	*Setophaga magnolia*	22	U	I	M	TP CP	
	Black-throated Green Warbler	*Setophaga virens*	10	C	I	M	TP CP	
	Rufous-capped Warbler	*Basileuterus rufifrons*	56	U	I	R	TP CP SM	P
	Golden-crowned Warbler	*Basileuterus culicivorus*	10	U	I	R	TP	P
	Wilson’s Warbler	*Cardellina pusilla*	30	U	I	M	TP CP	
	Slate-throated Whitestart	*Myioborus miniatus*	8	C	I	R	TP SM	
Cardinalidae	Western Tanager	*Piranga ludoviciana*	8	C	F/I	M	TP CP	
	White-winged Tanager	*Piranga leucoptera*	28	C	F/I	R	TP CP SM	
	Red-crowned Ant Tanager	*Habia rubica*	15	U	F/I	R	TP	
Thraupidae	Red-legged Honeycreeper	*Cyanerpes cyaneus*	70	C	F/I	R	TP CP SM	
	Cinnamon-bellied Flowerpiercer	*Diglossa baritula*	4	S	F/I	R	TP CP	

**Notes:**

* C, canopy; U, understory; S, open scrub; T, trunk.

** I, obligate insectivore; F/I, generalist insectivore that also eats fruit (omnivore); N/I, generalist insectivore that also eats nectar.

^ M, Nearctic-Neotropical migrant present in dry season; R, year-long resident; AM, Austral migrant present in wet season.

# B, Brewer & Christie (2020); DE, Dema (2020); D, Dietsch (2003); and P, Philpott et al. (2009).

^^ Production indicates which type of coffee systems the birds were sampled in where TP, traditional polyculture, CP, commercial polyculture, and SM, shaded monoculture.

We used liquid inserted into bird stomachs to induce regurgitation and stomach flushing. In 2001, we administered a 1% emetic solution of antimony potassium tartrate per 100 g of body mass (Poulin, Lefebvre & McNeil, 1994). Immediately after capture, we gave birds 0.8 cc per 100 g of body mass, although the amount administered was reduced to 0.6 cc per 100 g of body mass starting in June 2001 because it was sufficient to induce regurgitation. In 2002 and 2003, we used between 2–12 ml (depending on the size of the bird) of unflavored Pedialyte (water, dextrose, and <2% of potassium citrate, salt, sodium citrate, citric acid, and zinc gluconate) as rehydration with electrolytes is recommended for dehydrated songbirds (Welte & Miller, 2019). Some authors have suggested that stomach flushing is an invasive procedure for birds (*e.g.*, Zach & Falls, 1976, Johnson et al., 2002, Carlisle & Holberton, 2006). However, birds in our study experienced few obvious deleterious effects while being handled. One bird died (of >900 birds processed), but ~95% of processed birds flew away immediately after processing and some individuals were recaptured (but not processed) on subsequent sample days or seasons; other birds required <5–15 min to recuperate before flying away. During all years, we introduced liquid into bird stomachs with a lubricated catheter tube inserted into a 1.0-cc or 0.5-cc syringe. We used two different sizes of tubes (5 Fr and 8 Fr feeding tubes; Kendall Company, Mansfield, MA, USA) and selected tubes based on bird size. We also collected any fecal matter left in bird-processing bags and added this to stomach content samples. We transferred stomach content and fecal samples to vials filled with 96% ethanol with a micro-spatula and stored vials for arthropod identification. Vials were stored for 1 to 2 years before identification. We clipped the tip of the outer tail feathers to assure the same individual was not processed twice. We released birds immediately after processing.

We identified all captured birds to species, and later classified birds based on migratory status, *i.e*., year-round residents, Nearctic-Neotropical migrants at the study site during the dry season, or Austral migrants that breed at the study site during the wet season (Billerman et al., 2020). We also classified birds by foraging guild, *i.e*., obligate insectivore, generalist insectivore that also eats fruit (also known as omnivores), or generalist insectivore that also eats nectar based on previously used classifications from data collected in Neotropical coffee farms (Greenberg, Bichier & Sterling, 1997). Finally, we classified species based on their primary foraging strata in coffee agroecosystems, including canopy, open scrub (birds that forage in shrubs in open areas), trunk, and understory (Greenberg, Bichier & Sterling, 1997). Only species with at least four individuals sampled were included in our analysis.

### Arthropod identification

Arthropods were identified by two individuals (SML and MLB). We searched for whole bodies as well as body parts to aid in identification (*e.g.*, heads, mandibles, elytra, and wings). Arthropods were identified to order, superfamily, family, or genus as allowed by either whole bodies or body parts. We counted the number of individuals of each family or genus per sample if we viewed entire arthropod bodies or by reconstructing the number of individuals based on combinations of component body parts. Arthropods and arthropod parts with harder exoskeletons (*e.g.*, mandibles, heads, and elytra) are more likely to be preserved and identified from stomach content samples whereas soft-bodied arthropods such as spiders (Araneae), flies (Diptera), and butterflies and moths (Lepidoptera) may be less likely to be preserved and harder to identify from stomach samples (Rosenberg & Cooper, 1990; Sherry et al., 2016).

### Data analysis

To examine possible differences in diet composition, we performed permutational ANOVAS (PERMANOVAs) with the multivariate statistical software package PRIMER-E 7.0.13 (Clarke, 1993; Quest Research Limited, 2017). Similar to other studies visually identifying prey from avian insectivores (*e.g.*, Poulin & Lefebvre, 1996; Sherry et al., 2016), we classified dietary items into the following groups: Araneae, Auchenorrhyncha, Coleoptera, Diptera, Formicidae, Heteroptera, Other Hymenoptera (other than Formicidae), Lepidoptera, Orthoptera, and Infrequent (including infrequently encountered items such as Acari, Psocoptera, Sternorrhyncha, and Thysanoptera). We square-root transformed abundance of dietary items in each bird sample before analysis then created a Bray–Curtis similarity resemblance matrix and used PERMDISP (within PRIMER-E) to test each factor for homoscedasticity.

We ran a PERMANOVA where season, year, guild, and migratory status (resident or migrant) were fixed variables. To determine if diets of Nearctic-Neotropical migrants and residents differed during the dry season, we selected only dry season data and ran a PERMANOVA with year, guild, and migratory status as fixed variables. To determine if resident birds foraged differently in the dry and wet seasons, we selected only resident birds and ran a PERMANOVA with year, season (Dry or Wet), and foraging guild as fixed variables. To determine if coffee farm management affected avian foraging, we analyzed data for all species sampled across the three production systems with at least two samples in each production type (*N* = 4) during the wet season of 2001 when SM was well sampled. We ran a PERMANOVA with production management (CP, TP, and SM) as a fixed effect and bird species as a random effect.

PERMANOVAs were run for 999 permutations. Pseudo-*F*s and permutational *P* values are reported in the text. Terms found to be significant with the main PERMANOVA model were evaluated with pair-wise comparisons where pseudo-*t* values are reported. We used the SIMPER procedure (in PRIMER-E) to analyze individual contributions to significant terms.

## Results

We analyzed the stomach contents of 937 individuals representing 38 species, including 13 Nearctic-Neotropical migrants, one Austral migrant, and 24 year-round residents (Table 1). Overall, Coleoptera (mean = 1.62 ± 1.86 SD) and Formicidae (mean = 1.24 ± 5.23 SD) were the most frequently consumed prey per stomach sample. Samples contained a maximum number of 30 Acari, 24 Hymenoptera (other than Formicidae), 15 Auchenorrhyncha, 14 Coleoptera, and a stomach sample from one Swainson’s Thrush (*Catharus ustulatus*) contained 106 ants (Formicidae) (species-specific dietary components are provided in Table S2). Samples averaged 5.5 (+/−6.6 SD) dietary items each without differences between observers (SML averaged 5.3 +/− 6.9 SD species per sample and MLB averaged 6.5 +/− 4.7 SD). No species of coffee pests were identified in stomach contents.

The factors of year, guild, and season all showed homoscedasticity, but migratory status (*F* = 9.2, *P* = 0.002) and strata (*F* = 14.7, *P* = 0.001) did not, so caution is needed when interpreting the effects of these factors. Foraging guilds differed in consumption of prey from year to year and season to season (significant year × foraging guild × season interaction) (Pseudo *F* = 1.9, *P* = 0.036). Diet comparisons of resident and Nearctic-Neotropical migrant birds during the dry season when both were present revealed a significant year × guild × migratory status interaction (Pseudo *F* = 2.1, *P* = 0.02).

**Figure 1:**
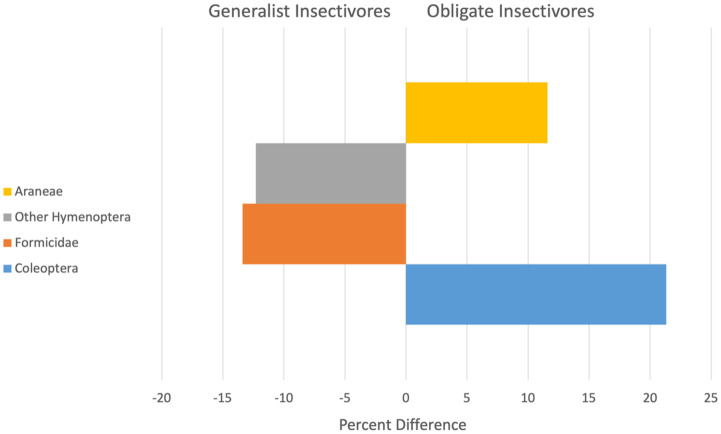
Arthropod taxa most responsible for contributing to significant differences (x-axis is Percent difference) in the diets of resident generalist insectivores (left) and obligate insectivores (right). Bars are arranged from highest contributor to the dissimilarity score at the base and include data for all study years.

Diets of obligate and generalist resident birds differed during some seasons and years, but not universally. Resident birds showed a significant season by year (Pseudo *F* = 1.9, *P* = 0.04) and year by guild (Pseudo *F* = 3.0, *P* = 0.002) interaction effect. Residents classified as obligate or generalist insectivores consumed significantly different prey items during the wet seasons of 2001 and 2002 and during the dry seasons of 2002 and 2003 (Table 2). Across all years, obligate or generalist insectivore diets of resident birds were ~72% dissimilar, with obligate insectivores consuming more beetles (Coleoptera) and spiders (Araneae) and generalist insectivores relying more on ants (Formicidae) and Other Hymenoptera (Fig. 1).

**Table 2:** Pair-wise comparisons shown to determine differences in the dietary components between resident birds categorized into obligate and generalist insectivore avian guilds.

Year	Wet season residents		Dry season residents	
	Pseudo-*t*	*P* (perm)	Pseudo-*t*	*P* (perm)
2001	**1.8**	**0.015**	1.2	0.22
2002	**1.9**	**0.003**	**1.9**	**0.004**
2003	1.5	0.068	**2.0**	**0.002**

**Note:**

Comparisons are divided by season and year. Pseudo-*t* and permutational *P* values are reported for 2001–2003, with statistically significant results in bold font.

**Figure 2:**
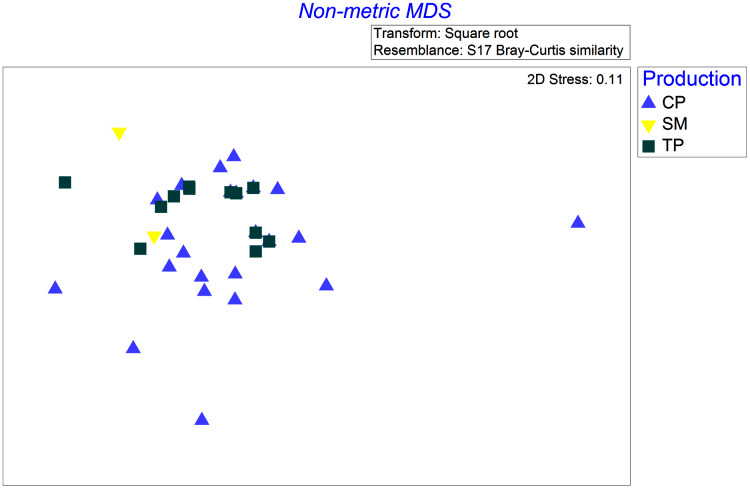
Non-metric multi-dimensional scaling ordination based on Bray-Curtis dissimilarities of square root transformed abundances of arthropod taxa identified in stomach contents of Yellow-Green Vireos (*Vireo flavoviridis*) foraging in Commercial Polyculture (CP), Shaded Monoculture (SM), and Traditional Polyculture (TP) coffee systems.

Four species were sampled in the wet season of 2001 with at least two samples in each of the coffee production systems (TP, CP, and SM): Rufous-capped Warbler (*Basileuterus rufifrons*), Spot-breasted Wren (*Pheugopedius maculipectus*), White-winged Tanager (*Piranga leucoptera*), and Yellow-green Vireo (*Vireo flavoviridis*). These four species differed in how their diets changed across productions systems (significant production × bird species interaction, Pseudo *F* = 1.6, *P* = 0.046). Differences in coffee production management were associated with differences in the diets of Yellow-green Vireos, but not the other three species. Diets of Yellow-green Vireos (YGVI) differed significantly between CP and TP (61% dissimilar, *t* = 1.5, *P* = 0.04), but not strongly enough between either CP and SM (77.8% dissimilar, *t* = 1.6, *P* = 0.07) or TP and SM (65% dissimilar *t* = 1.8, *P* = 0.058). Yellow-green Vireos are classified as generalist insectivores that often forage in the canopy. In traditional polyculture systems, YGVI consumed more beetles (Coleoptera) than they did in commercial polyculture systems or shaded monoculture, accounting for 25% and 36% of the dietary pairwise differences respectively. In shaded monoculture YGVI consumed more ants (Formicidae) accounting for ~36% of the dietary pairwise differences between SM and both CP and TP systems (Fig. 2).

## Discussion

### Diet descriptions

Our first research objective was to identify the major arthropod prey in the diets of obligate and generalist insectivorous birds. Among resident birds, those that were obligate insectivores consumed more spiders (Araneae) and beetles (Coleoptera), whereas generalist insectivores consumed more ants (Formicidae) and Other Hymentoptera prey that may be easier prey to find due to clumped distributions (*e.g.*, Johnson, 2000; Sherry et al., 2016). Furthermore, Formicidae may be lower quality prey because of their chemical defenses (Eisner, Eisner & Siegler, 2005) and low nutritional value (Zach & Falls, 1978). All arthropod taxa documented in the diets of 30 of 39 bird species in our study (Araneae, Auchenorrhyncha, Coleoptera, Diptera, Formicidae, Heteroptera, Other Hymenoptera, and Lepidoptera) have also been reported as being important prey in other studies conducted in the Neotropics (*e.g.*, Poulin & Lefebvre, 1996; Sherry et al., 2016). Notable is that several foraging studies document removal of these same arthropods solely from foraging observations (*e.g.*, Wunderle & Latta, 1998; Jones et al., 2000; Newell et al., 2014). Moreover, excluding birds from coffee plants and shade trees in coffee farms sometimes leads to reduction in many of these same taxa compared to control plants to which birds have access (Greenberg et al., 2000; Philpott et al., 2004), but this is not always the case (Johnson et al., 2009; Karp & Daily, 2014). These results thus confirm some of what we know about the diets of insectivorous birds and emphasize the need for direct measurement of diet items, rather than inferences from bird foraging or removal experiments.

### Annual variation

Our second research objective was to determine how diets might change over time or with season, and we found significant annual and seasonal differences in bird diets. We found that bird foraging guilds differed in their consumption of prey taxa from year to year and the diets of resident birds differed by guild and year. In general, significant annual effects on diets of insectivorous birds may reflect annual differences in resource availability, fluctuating precipitation, and changing migration patterns (McNamara et al., 2011; Studds & Marra, 2011). At our sites, differences in the diets of migrants and residents in the dry season were most apparent in 2003, and this was the same year we observed differences in the diets of Austral migratory YGVI and resident birds in the wet season. Differences in the diets of migrants and residents may be illustrative of behavioral adaptation to the seasonal changes in avian densities where resource competition may fluctuate annually in response to ecological conditions, such as changes in arthropod populations resulting from high precipitation (Keast & Morten, 1980; Greenberg, 1995). More specifically, shifts in avian diets over time are likely an opportunistic adaptation by both obligate and generalist insectivores to seasonal fluctuations in prey availability that may be exacerbated by wet and dry years of the ENSO cycle (Sillett, Holmes & Sherry, 2000). Significant differences between resident obligate and generalist insectivores in the wet seasons of 2001 and 2002 were absent in 2003 which may be due to precipitation differences. Precipitation data indicate that 2003 was an abundantly wet year when 3,446 mm of precipitation fell in Tapachula municipality compared to 3,057 mm in 2002 (Thornton et al., 2017). During years of less rainfall, arthropod populations may be reduced and resource competition for food resources among insectivorous birds may be high (*e.g.*, Tilman, 1982); in wetter years, greater prey abundance may mitigate competition from interspecific dietary overlap (Sherry et al., 2016).

Although annual effects were important, diets of Nearctic-Neotropical migrants and residents were dissimilar across all years but unequal homogeneity of migrant vs resident dispersions means that we cannot confirm these differences are a result of the groups foraging differently (Warton, Wright & Wang, 2012). Poulin & Lefebvre (1996) reported little overlap in the diets of migrants and residents in Panamanian forests, specifically quantified as statistically higher consumption of Hymenoptera (ants), Coleoptera, Isoptera, Diplopoda, insect larvae, Gastropoda, Chilopoda, and smaller (0–10 mm) prey among Nearctic-Neotropical migrants and more Hymenoptera (alate ants), insect pupae, lizards, Heteroptera, Orthoptera, and Araneae, and larger (>25 mm) prey among resident birds. Further, Boyle, Conway & Bronstein (2011) found that migrants were more likely to be generalist insectivores and rely on fruit than tropical residents; a change to a more frugivorous diet might alter demand by migrant birds for particular taxa of insect prey.

### Seasonality

In 2002 and 2003, diets of obligate and generalist insectivore residents differed during the dry season, but not during the wet season, and the reverse was true in 2001. In addition, season was an important factor in our PERMANOVA interacting with year and guild to influence avian diets. Seasonality of food resources and breeding requirements likely interact, so whereas generalist insectivores may consume fruits and seeds, they may need to prioritize certain arthropod taxa during the breeding season for reproductive requirements (Gill, Prum & Robinson, 2019), potentially increasing dietary overlap with obligate insectivores. An exception to this general trend is the YGVI, the Austral migratory species, that consumes fruit and insects during the wet season while breeding at our study sites (Brewer & Christie, 2020).

Unfortunately, we do not have consistent arthropod data from all seasons when bird diets were studied to compare the relative abundance in the farm to relative abundance in the diets. In general, differences in foraging behavior (Greenberg et al., 1997, 1999) and vegetative strata (Whelan, 2001; Jedlicka et al., 2006) are important factors that influence diet selection.

### Shade management and ecosystem services

Our third objective was to determine how diets might be influenced by coffee agroecosystem management. Differences in diets were evident, with one-fourth of bird species in all three production systems having significantly different diets in both commercial polyculture and traditional polyculture systems. Although we did not measure fruit availability, other investigators have suggested that differences in diet in the same farms may be due to the greater availability of fruit in the traditional polyculture (Dietsch, Perfecto & Greenberg, 2007), which may have bottom-up effects on arthropod composition or result in a separate change in the arthropod communities that may affect prey availability and influence selection. With greater vegetational diversity, traditional polyculture systems may have a greater diversity and abundance of natural predators (Letourneau et al., 2009; Serrano-Davies & Sanz, 2017), leading to increased availability of spiders and presence in avian diets. Since MacArthur (MacArthur, 1958), differences in diet have been identified as pathways to coexistence of insectivorous species of birds over time and, indeed, we found evidence of dietary niche partitioning in our study. Shade management affected the diets of Yellow-green Vireos (YGVI) because arthropod composition of their diets differed depending on whether they were foraging in TP, CP, or SM. YGVI consumed more Coleoptera in TP and more Formicidae in SM systems. Nevertheless, the diets of the other three species sampled across all shade management systems did not change significantly across management systems. This is likely a result of low sample sizes across all systems. However, it may be true that generalist insectivores may not be as impacted by production changes as obligate insectivores. Some bird species may alter their foraging patterns in response to fruit resources or management shifts in coffee farms (Carlo, Collazo & Groom, 2004). Other investigators have documented differences in where and how birds forage in shaded coffee plantations (Jones et al., 2000; Bakermans et al., 2012), and that even small differences (*e.g.*, tree height and composition) may influence the foraging behavior and diets of some species (*e.g.*, Dietsch, Perfecto & Greenberg, 2007; Newell et al., 2014). To fully understand the implications of management shifts on bird foraging and diets, more studies are needed that explicitly link prey availability, species-specific foraging observations, and dietary analysis; such studies would help parse out cause and effect relationships.

The effect of habitat management is a vital consideration for understanding pest control ecosystem services birds may provide in agricultural landscapes (Perfecto et al., 2004; Van Bael et al., 2008; Jedlicka, Greenberg & Letourneau, 2011; Iverson et al., 2014; Milligan et al., 2016; Şekercioğlu, Wenny & Whelan, 2016). The effects of birds on populations of coffee pests are often more efficient in coffee agroforests with more diverse shade (Perfecto et al., 2004; Johnson, Kellermann & Stercho, 2010; Milligan et al., 2016). Mechanisms driving this enhanced pest control could be increases in taxonomic or functional richness of the bird community, or increased abundance of insectivores (Perfecto et al., 2004; Van Bael et al., 2008; Philpott et al., 2009; Philpott & Bichier, 2012; Milligan et al., 2016; Martínez-Salinas et al., 2016). In addition, differences in bird foraging behavior that result from differences in tree species, composition, or foraging strata present may underlie differences in bird diets (Wunderle & Latta, 1998; Dietsch, Perfecto & Greenberg, 2007; Bakermans et al., 2012; Newell et al., 2014) and, therefore, the effectiveness of bird predation on pest species in coffee agroecosystems. For this reason, we assessed if birds consumed coffee pests, or if their diets, generally, shifted with management or season. In surprising contrast to previous studies, we found no arthropod pest insects in the diets of the birds sampled. Specifically, coffee berry borers (*Hypothenemus hampei*; 1.2–1.8 mm long) found in the diets of migratory wood warblers (Parulidae) in Jamaican coffee farms (Sherry et al., 2016) were noticeably absent in our study. Data collected during our study indicates that coffee berry borer abundance and fruit infestation rates were low. In vacuum samples of insects from coffee plants obtained in February 2001 and August 2001, borers comprised only 10 of 835 (1.2%) of insects collected (S. Philpott, 2001, unpublished data). In addition, only ~6% of sampled fruits were found to be infested by coffee berry borers between November 2000 and June 2001 (S. Philpott, 2001, unpublished data). In other studies, borers have been described as abundant prey items (*e.g.*, Sherry et al., 2016). Notably, the role of insectivorous birds (and bats) in limiting arthropods and coffee damage may also shift depending on landscape context (Librán-Embid, De Coster & Metzger, 2017). More work is needed to understand the impacts of coffee landscape change on bird diets, foraging, and pest predation.

### Methods for diet analysis and conservation implications

Although questions about how to best study and analyze bird diets are not new (Rosenberg & Cooper, 1990), alternative methods provide complementary insights into the arthropod prey consumed by birds. Molecular scatology provides more specific identification of prey items, often to genus or species-level, but has been criticized for failing to account for individuals and relying on presence/absence data for analysis (Symondson & Harwood, 2014; but see Deagle et al., 2019). Compared to molecular techniques, either stomach-content studies using emetics (*e.g.*, Sherry et al., 2016) or fecal dissection approaches (*e.g.*, Burger et al., 1999) allow researchers to reliably quantify prey items. Admittedly emetics and fecal dissection recover more hard-bodied prey items (such as Coleoptera and Formicidae), but these biases extend across all samples and allow comparisons to be made. Some drawbacks of using emetics include potential to harm birds not only during sample collection, but after release (Johnson et al., 2002). Ethically, it is important to consider avoiding more invasive procedures and substituting non-invasive procedures such as fecal collection that can be used easily in molecular scatology. Studies using emetics have generally gathered quite different quantities of arthropods identified per stomach sample (5.5 in this study compared to 34 in Kent & Sherry (2020), 30 in Strong (2000) and 70 in Sherry et al. (2016)). These differences most likely reflect changes in the arthropod communities at different study sites. All three of the aforementioned studies took place in the tropical island of Jamaica, compared to this study that took place in the mountains of Southern Mexico. Bird diets will obviously be constrained by resource availability.

Finally, both emetic and fecal dissection lead to dietary items that are identified at a courser level, oftentimes to order, leading to analyses that may overestimate similarity between samples (Anderson et al., 2005). The fact that we found such striking differences between samples using course dietary categories reaffirms the inherent differences between samples in our study. However, such a course overview of diets may gloss over important distinctions of prey identity and the ecological interactions behind predator-prey dynamics. Species-level identification is necessary to understand if predators consume certain pests, for example. Because taxonomic resolution can influence results, combining molecular scatology methods with visual identification of stomach/fecal matter can provide a more comprehensive view of diets. Increasing sample sizes and combining studies from across species’ ranges will enhance our knowledge of avian diets and lead to better management for conservation and predictions of the role birds play in community-level interactions. Studies that provide baseline dietary data of avian insectivores are crucial for evaluating how community dynamics change as a result of the global decline in terrestrial insect populations (Klink et al., 2020). Although diet studies are complicated and logistically difficult, they are crucial for monitoring and conserving bird populations in the future.

## Conclusions

Our study shows that obligate and generalist insectivorous birds differed in their most consumed prey taxa, with generalist birds relying on lower quality prey items. We found that bird diets also differed among migrants and residents, between wet and dry seasons, and from year to year. Human management also influenced Yellow-green Vireo diet selection with more ants being consumed in more intensively managed Neotropical coffee systems. Although bird diets vary, understanding important prey items is fundamental natural history knowledge that can inform conservation efforts and evaluate habitat concerns.

## Supplemental Information

10.7717/peerj.12296/supp-1 Supplemental Information 1 Vegetation differences in coffee agroecosystem sites where birds diets were assessed between January 2001–March 2003. Sites are listed in order of management intensity from the least to the most intensive management.

10.7717/peerj.12296/supp-2 Supplemental Information 2 Percentage of arthropod dietary components identified to order, superfamily or family and total number of individual arthropods identified per bird species.

10.7717/peerj.12296/supp-3 Supplemental Information 3 Raw data.

## Additional Information and Declarations

### Field Study Permissions

The following information was supplied relating to field study approvals (*i.e*., approving body and any reference numbers): Mexico’s Secretaría de Medio Ambiente y Recursos Naturales approved this study (Permit Number 10092).

### Data Availability

The following information was supplied regarding data availability: Raw data are available in the Supplemental Files.
