# Supplementary material for: Differences in insectivore bird diets in coffee agroecosystems driven by obligate or generalist guild, shade management, season, and year
Source: PeerJ. 2021 Oct 27;9:e12296. doi: 10.7717/peerj.12296 (PMC8556712; doi:10.7717/peerj.12296)
Supplement: Supplemental Information 1 — Sites are listed in order of management intensity from the least to the most intensive management. [file peerj-09-12296-s001.docx]

**Appendix Table 1.** Vegetation differences in coffee agroecosystem sites where birds diets were assessed between January 2001-March 2003. Sites are listed in order of management intensity from the least to the most intensive management.

|  |  | **Mas & Dietsch (2003)^¶^** | | | | **Philpott et al. 2006^§^** | | |
| --- | --- | --- | --- | --- | --- | --- | --- | --- |
| **Site Name** | **Site Code** | **No. tree species** | **No. trees** | **Canopy Cover** | **MI*** | **No. tree species** | **Canopy cover** | **MI*** |
| Belen Rustic | TP1 | 7.3 | 9.8 | 92.3% | 2.02 | 14 | 73.5% | 2.19 |
| Irlanda Restoration | TP2 | 6 | 10.5 | 54.3% | 4.27 | NA | NA | NA |
| Irlanda Production | CP2 | 3.8 | 8.8 | 58.8% | 4.5 | 7.3 | 65.7% | 2.88 |
| Belen Production | CP1 | 4.3 | 8 | 48.2% | 5.08 | 6.5 | 50.7% | 3.6 |
| Hamburgo | SM | 2 | 4.3 | 14.5% | 5.71 | 3 | 33.0% | 4.07 |

^¶^In this study, vegetation was sampled in four randomly selected, 12 m radius (or 452.16 m^2^) points in each site during June and July of 1998. All values shown are mean values for each site.

^§^In this study, vegetation was sampled in ten (CP2 and SM) or six (TP1 and CP1) 35 x 35 m (or 1225 m^2^) plots surrounding bird exclosures placed in each site in November 2000 and January 2003. All values shown are mean values for each site.

*The Management Index (MI) is a scaled mean of vegetation variables where values of individual factors (e.g., tree richness, canopy cover) are scaled from 0 (highest value across all points) to 1 (lowest across all points) and then averaged. The MI was calculated in the same way in both studies. A higher MI reflects greater agricultural management intensity.
